# The *dif*/Xer Recombination Systems in Proteobacteria

**DOI:** 10.1371/journal.pone.0006531

**Published:** 2009-09-03

**Authors:** Christophe Carnoy, Claude-Alain Roten

**Affiliations:** 1 INSERM U801, Lille, France; 2 Univ Lille Nord de France, Lille, France; 3 UDSL, Faculté des Sciences Pharmaceutiques et Biologiques, Lille, France; 4 Department of Fundamental Microbiology (DMF), Biology Building, Biophore, Quartier de la Sorge, Lausanne University, Lausanne-Dorigny, Switzerland; 5 Institute of Microbiology (IMUL), University Hospital Center of Lausanne (CHUV), Lausanne University, Lausanne, Switzerland; University of Hyderabad, India

## Abstract

In *E. coli*, 10 to 15% of growing bacteria produce dimeric chromosomes during DNA replication. These dimers are resolved by XerC and XerD, two tyrosine recombinases that target the 28-nucleotide motif (*dif*) associated with the chromosome's replication terminus. In streptococci and lactococci, an alternative system is composed of a unique, Xer-like recombinase (XerS) genetically linked to a *dif*-like motif (*dif*_SL_) located at the replication terminus. Preliminary observations have suggested that the *dif*/Xer system is commonly found in bacteria with circular chromosomes but that assumption has not been confirmed in an exhaustive analysis. The aim of the present study was to extensively characterize the *dif*/Xer system in the proteobacteria, since this taxon accounts for the majority of genomes sequenced to date. To that end, we analyzed 234 chromosomes from 156 proteobacterial species and showed that most species (87.8%) harbor XerC and XerD-like recombinases and a *dif*-related sequence which (i) is located in non-coding sequences, (ii) is close to the replication terminus (as defined by the cumulative GC skew) (iii) has a palindromic structure, (iv) is encoded by a low G+C content and (v) contains a highly conserved XerD binding site. However, not all proteobacteria display this *dif*/XerCD system. Indeed, a sub-group of pathogenic ε-proteobacteria (including *Helicobacter* sp and *Campylobacter* sp) harbors a different recombination system, composed of a single recombinase (XerH) which is phylogenetically distinct from the other Xer recombinases and a motif (*dif*_H_) sharing homologies with *dif*_SL_. Furthermore, no homologs to *dif* or Xer recombinases could be detected in small endosymbiont genomes or in certain bacteria with larger chromosomes like the Legionellales. This raises the question of the presence of other chromosomal deconcatenation systems in these species. Our study highlights the complexity of *dif*/Xer recombinase systems in proteobacteria and paves the way for systematic detection of these components in prokaryotes.

## Introduction

In bacteria, DNA replication of circular chromosomes can generate, by homologous recombination, concatenated chromosomes that affect cell viability. In *Escherichia coli*, resolution of chromosomal concatenates requires a site-specific recombination system involving two tyrosine recombinases (XerC and XerD) associated with FtsK, the DNA translocase involved in bacterial division [1], [2], [3]. Chromosomal deconcatenation occurs at a specific chromosome site referred to as *dif*, for the “deletion-induced filamentation”, a phenotype observed in *E. coli* strains which are either deficient in XerD or XerC recombinases or lack the *dif* sequence [4], [5]. The 28-nt *dif* locus is a palindromic motif composed of two inverted repeats (each of which is specifically targeted by one of the two Xer recombinases) separated by a central hexanucleotide. The *E. coli dif* sequence is located opposite the origin of chromosomal replication, i.e. near the chromosome terminus at the junction of oppositely polarized DNA sequence elements in a 30 kb-region called the *dif* activity zone (DAZ) [6], [7], [8], [9].

The Xer recombination system was originally described for *E. coli* plasmids [10], [11] but is not restricted to this bacterial species, since homologous systems have been functionally characterized in *Bacillus subtilis*, *Haemophilus influenzae*, *Xanthomonas campestris*, *Caulobacter crescentus* and *Vibrio cholerae* [12], [13], [14], [15], [16]. Xer-related recombinases have also been detected by sequence homology or DNA hybridization in many bacterial taxa and some archaeal species [17], [18], [19], [20]. Homologs to *E. coli dif* sequences have been found in other proteobacteria, firmicutes and actinobacteria [15], [21], [22], [23], [16], suggesting the universality of the *dif*/Xer system in the bacterial kingdom. Recently, an unconventional single Xer-like recombinase targeting an atypical *dif* sequence was described in streptococci and lactococci [23].

In addition to its role in chromosome dimer resolution, the *dif* locus may be involved in the integration/excision of exogenous DNA. For instance, the filamentous phages CTXΦ and VGJΦ in *Vibrio cholerae*, f237 in *V. parahaemoliticus*, CUS-1 in *E. coli* 018:K1:H7, YpfΦ in *Yersinia pestis* and Cf16-v1 and ΦLf in *Xanthomonas campestris* all integrate into the host chromosome at the *dif* site [24], [25], [26], [27], [28], [29], [30]. The mechanism of prophage genome integration has been described in detail in *V. cholerae* CTXΦ, the filamentous phage containing the cholera toxin-encoding gene [31], [32]. Recently, Val et al. showed that after appropriate folding, CTXΦ's single-stranded phage DNA forms a *dif*-like structure that irreversibly recombines with the bacterial *dif* by using host XerC and XerD recombinases [32]. This clearly demonstrates that *dif* is a preferential integration site for single-stranded filamentous phages displaying *dif*-like motifs on their genome. Other large genetic DNA elements also target the *dif* sequence, as evidenced by integration of the 57-kb gonococcal genetic island (GGI, containing a type IV secretion system) into the *Neisseria* chromosome [33], [34]. Taken as a whole, these studies strongly suggest that the *dif* sequence is a preferential site for exogenous DNA integration and thus contributes to genome evolution in general and to virulence gene acquisition in particular. Moreover, *dif*'s natural ability to integrate exogenous DNA has been used to deliver genes of biotechnological interest to the bacterial chromosome [35], [36].

Despite the publication of many bacterial genome sequences (440 had been sequenced as of January 1^st^, 2007) with over half of these from proteobacteria, no exhaustive analysis of the *dif*/Xer system has yet been undertaken. As the *dif* sites do not appear in GenBank's genome annotation, we developed a strategy for systematically identifying *dif*-related sequences in proteobacteria chromosomes by combining similarity search tools (BLAST and YASS) with genometric methods (cumulative GC-skew analyses). In contrast to initial assumptions in the literature, we found that not all proteobacterial circular chromosomes feature a *dif*/Xer system and that a ε-proteobacteria sub-group harbors an atypical *dif*/Xer system, indicating heterogeneity of this recombination system in proteobacteria. This report represents the first comprehensive analysis of the *dif* motifs and of their associated recombinases and should facilitate the identification of related recombination systems in prokaryotes.

## Results

### The *dif*-related sequences are highly conserved among the proteobacteria

To detect *dif* homologs in proteobacterial chromosomes, we developed an *in silico* approach based on (i) homology of the candidate with the experimentally characterized proteobacterial *dif* sequences in *E. coli*, *C. crescentus*, *X. campestris*, *V. cholerae* and *H. influenzae* or with a related sequence found in a close taxon, (ii) location of the putative sequence near the chromosome terminus, as defined by the cumulative GC skew analysis, (iii) presence in different strains of the same species, and (iv) presence of a single copy of the *dif* candidate within the chromosome. Using this strategy, 234 chromosomes from 156 proteobacterial species were analyzed (Table 1 and Table S1). *dif* homologs were found in 87.2% of the chromosomes (204 out of 234) and in 87.8% (137 out of 156) of the species. A *dif*-related sequence was detected in all β and δ-species and in 97.7% (44 out of 45) and 82.8% (53 out of 64) of the α and γ-species, respectively. Surprisingly, only 1 out of 8 ε–proteobacterial species (12.5%) harbored a *dif*-related sequence. Lastly, one unclassified proteobacterium (*Magnetococcus* sp.) also displayed a *dif* homolog.

**Table 1:** Genome and *dif* features of a representative panel of proteobacteria.

	Genome features			putative *dif* features				
Species	size (bp)	G+C content	Maximum CGC skew	Nucleotide sequence (1)	position on genome	G+C content	distance from GCG skew	intergenic location (2)
**α-proteobacteria**								
Caulobacterales								
* Caulobacter crescentus* CB15	4016947	0.672	1930040	AAGATCGACTTTG**T** AATTTATGTAAAGT	1946380	0.250	14159	yes
Rhizobiales								
* Agrobacterium tumefaciens* str. C58 chr. circular	2841490	0.593	1485983	TAATCGCATAAGA**T**ATATTATGGAACTT	1478815	0.250	7168	yes
* Bartonella quintana* str. Toulouse	1581384	0.387	724981	AAATTCCATAATA**T**ATATTATGCGATAA	720906	0.179	4075	yes
* Bradyrhizobium japonicum* USDA 110	9105828	0.640	4893406	GATTCGCATAAGG**T** ATATTATGGAATAT	4996172	0.286	102766	yes
* Brucella melitensis* 16M chr. I	2117136	0.571	955452	TAATCGCATAAGA**T** AGATTATGGAACTG	954740	0.321	712	yes
* Brucella melitensis* 16M chr. II	1177785	0.573	757557	AAATCAGATAATA **T**GTATTATGGAACAT	758183	0.214	626	yes
* Mesorhizobium loti* MAFF303099	7036071	0.627	203543	AAGTCGCATAAGA**T** AGATTATGGAACTT	299619	0.321	96076	yes
Rhodobacterales								
* Rhodobacter sphaeroides* 2.4.1 chr. 1	3188599	0.690	1435088	GAGTCGGATAATC**T**GTATTATGTATTCT	1436843	0.321	1755	yes
* Rhodobacter sphaeroides* 2.4.1 chr. 2	943016	0.690	399979	TTATCTGATAAGC **A**AGATTATGTAATCA	371575	0.250	28404	yes
Rhodospirillales								
* Magnetospirillum magneticum* AMB-1	4967148	0.650	2616185	CGTCGCCATAATA**T** ACATTATGCGACAA	2610339	0.393	5846	yes
Rickettsiales								
* Ehrlichia ruminantium* str. Welgevonden	1516355	0.274	766761	ATATTACATAATG**T** ATATTATGGAAAAT	747982	0.143	18779	yes
* Rickettsia prowazekii* str. Madrid E	1111523	0.290	628915	TTGTTCTATAATA **T**GTATTATGGAAAAT	596105	0.179	32810	yes
Sphingomonadales								
* Novosphingobium aromaticivorans* DSM 12444	3561584	0.652	2030402	AGGATTGATAATA**A** TCATTATGTAAATA	2048172	0.179	17770	yes
**β-proteobacteria**								
Burkholderiales								
* Bordetella pertussis* Tohama I	4086189	0.677	2227724	AATTCGCATAATG**T** ATATTATGTAAAGT	2229069	0.214	1345	yes
* Burkholderia mallei* ATCC 23344 chr. 1	3510148	0.681	1086094	AATGTCGATAATT**G**ATATTATGTCAAAT	1081309	0.214	4785	hyp. prot
* Burkholderia mallei ATCC* 23344 chr. 2	2325379	0.689	1077185	AATGTCGATAATT**T**GCGTTATGTCAAAT	1075135	0.286	2050	yes
* Ralstonia solanacearum* GMI1000	3716413	0.670	2009173	CCATCGCATAATT**T** ATCTTATGTTAAAT	2031219	0.250	22046	yes
* Rhodoferax ferrireducens* DSM 15236	4712337	0.598	2485317	ACTTGATACGATG**T** ATATTATGTTAAGT	2472550	0.250	12767	yes
Hydrogenophilales								
* Thiobacillus denitrificans*	2909809	0.660	1440104	ACTTCGCATAATG**T** ATATTATGTTAAAT	1430783	0.214	9321	yes
Methylophilales								
* Methylobacillus flagellatus* KT	2971517	0.557	1573478	ACTTCGCATAATG**T** ATATTATGTAAAAT	1564653	0.214	8825	yes
Neisseriales								
* Neisseria meningitidis* MC58	2272351	0.515	1231577	AGTTCGCATAATG**T** ATATTATGTTAAAT	1229349	0.214	2228	hyp. prot
Nitrosomonadales								
* Nitrosomonas europaea* ATCC 19718	2812094	0.507	964528	ATTTCGTATAATG**T** ATATTATGTTAAAT	974219	0.143	9691	yes
Rhodocyclales								
* Dechloromonas aromatica* RCB	4501104	0.592	2186143	AACGCGCATAATT**T**GCATTATGTTAAAT	2192508	0.286	6365	yes
**δ-proteobacteria**								
Bdellovibrionales								
* Bdellovibrio bacteriovorus* HD100	3782950	0.506	1940732	TCTTCTGATAAGT**T** ATATTATGTAACGG	1946858	0.286	6126	yes
Desulfobacterales								
* Desulfotalea psychrophila* LSv54	3523383	0.468	2260306	TAAGGAGATAAAT **T**GATTTATGAAAACG	2338241	0.250	77935	yes
Desulfovibrionales								
* Desulfovibrio vulgaris* subsp. Vulgaris str. Hildenborough	3570858	0.631	1735879	ATGTCCCATAATG**T** AAATTATGTTAACT	1754277	0.250	18398	yes
Desulfuromonadales								
* Geobacter sulfurreducens* PCA	3814139	0.609	1865942	ACGTCCCATAAGA**T**ATATTATGTAAAGT	1891880	0.286	25938	yes
Myxococcales								
* Anaeromyxobacter dehalogenans* 2CP-C	5013479	0.749	1906268	ACGTCCGATAATA**T**GGATTATGGTAACT	1906477	0.357	209	yes
* Myxococcus xanthus* DK 1622	9139763	0.688	4547166	AGGTCCGATAACA**T**GCGTTATGTAAACT	4489697	0.393	57469	yes
Syntrophobacterales								
* Syntrophus aciditrophicus* SB	3179300	0.514	1665473	TTGTCCTATAAGA**T**ATATTATGTAAACC	1665861	0.250	388	yes
**ε-proteobacteria**								
Campylobacterales								
* Sulfurimonas denitrificans* DSM 1251 (3)	2201561	0,34	1135161	TTTCAATAGAATT**T** ACATTATGTTAACC	1122264	0.175	12897	yes
**γ-proteobacteria**								
Aeromonadales								
* Aeromonas hydrophila* ATCC7966	4744448	0.615	2494705	ACCGCGCATAATG**T** ATATTATGTTAAAT	2514936	0.286	20231	yes
Alteromonadales								
* Idiomarina loihiensis* L2TR	2839318	0.470	1411650 (4)	ATTGCGTATAATG**T** ATATTATGTTAAAT	1387623	0.179	24027	yes
* Shewanella oneidensis* MR-1	4969795	0.459	2490130	ACTGCGCACAATG**T** ATATTATGTTAAAT	2476928	0.286	13202	yes
Chromatiales								
* Nitrosococcus oceani* ATCC 19707	3481691	0.503	1849931	TGTTCGCATAATA **C**ATATTATGTTAAAT	1850410	0.214	479	yes
Enterobacteriales								
* Erwinia carotovora* subsp. atroseptica SCRI1043	5064019	0.509	2552458	GGTTCGCATAATG**T** ATATTATGTTAAAT	2532133	0.250	20325	yes
* Escherichia coli* K12	4639675	0.507	1549688	GGTGCGCATAATG**T** ATATTATGTTAAAT	1588788	0.286	39100	yes
* Sodalis glossinidius* str. ‘morsitans’	4171146	0.546	2461688	AGTACGCATAATG**T** AGATTATGTTAAAT	2471148	0.250	9460	yes
* Yersinia pestis* CO92	4653728	0.476	2562641	GGTGCGCATAATG**T** ATATTATGTTAAAT	2562919	0.286	278	yes
Methylococcales								
* Methylococcus capsulatus* str. Bath	3304553	0.635	1531625	TATGCGCATAATG**T** ATATTATGTTAAAT	1492525	0.214	39100	yes
Oceanospirillales								
* Hahella chejuensis* KCTC 2396	7215267	0.538	3439027	AGTGCGCATAATA**T**ATATTATGTTAAAT	3437061	0.214	1966	yes
Pasteurellales								
* Haemophilus influenzae* Rd KW20	1830023	0.381	1474989	ATTTCGCATAATA**T** AAATTATGTTAAAT	1473975	0.143	1014	yes
Pseudomonadales								
* Acinetobacter* sp. ADP1	3598621	0.404	1847121	GATTCGTATAATG**T** ATATTATGTTAAAT	1848733	0.179	1612	yes
* Pseudomonas aeruginosa* PAO1	6264403	0.665	2428120	GATTCGCATAATG**T** ATATTATGTTAAAT	2443082	0.214	14962	yes
Thiotrichales								
* Francisella tularensis* subsp. tularensis Schu 4	1892819	0.322	950050	CATTCGTATAATA**T**ATATTATGTTAAAT	994689	0.143	44639	yes
Vibrionales								
* Vibrio cholerae* O1 biovar eltor str. N16961 chr. I	2961116	0.476	1564264	AGTGCGTATTATG**T** ATGTTATGTTAAAT	1564118	0.250	146	yes
* Vibrio cholerae O1* biovar eltor str. N16961 chr. II	1072311	0.469	512448	AATGCGCATTACG **T**GCGTTATGTTAAAT	507996	0.357	4452	yes
Xanthomonadales								
* Xanthomonas campestris* pv. campestris str. ATCC 33913	5076172	0.650	2442019	TCCTGACATAATA**T** ACATTATGCGAAAT	2441762	0.286	257	yes

(1) The central nucleotide in bold defines the position of the *dif* sequence on the chromosome. The nucleotides involved in the palindrome are underlined.

(2) hyp.prot.  =  *dif* inserted into a hypothetical protein-encoding gene.

(3) Sulfurimonas denitrificans strain DSM 1251  = Thiomicrospira denitrificans ATCC 33889.

(4) maximum of the GC skew.

**Figure 1:**
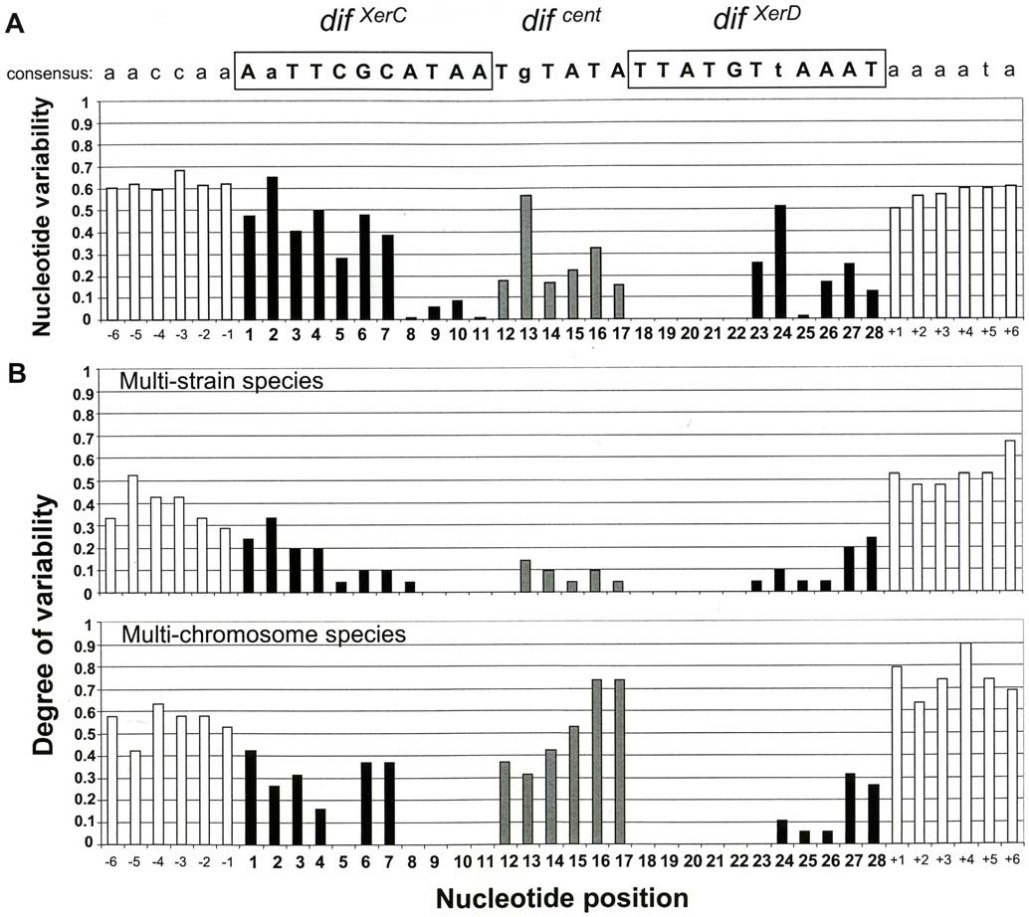
Nucleotide variability within *dif*-related sequences. (A) Consensus sequence and *dif* nucleotide variability for 161 *dif*-related sequences from 137 proteobacterial species. Nucleotide sequence characters in bold represent the *dif* sequence (28-mer). If the nucleotide frequency represents more than 50%, it is written in upper case letters; if not, the nucleotide is written in lower case letters. The nucleotide variability at each position in the 28-mer was defined as 1–*f*, where *f* is the frequency of the most frequent nucleotide. Nucleotide frequencies at each position are given in Table S2. Black bars represent *dif* ^XerC^ and *dif* ^XerD^ nucleotides, whereas grey bars correspond to the the *dif* ^cent^ nucleotides. White bars represent nucleotides outside *dif*. (B) Degree of variability in the *dif* sequence in 21 multi-strain species and in 19 multi-chromosome species. The degree of variability was calculated for each nucleotide position, as described in the Methods section.

To avoid redundancy, the first-published chromosome sequence in a species was considered to be representative. Thus, of the 204 *dif* sequences that we characterized, 161 were considered to be representative of the different proteobacterial taxa and were therefore used to define a consensus sequence (Figure 1 and Table S2). The two undecanucleotides (11-mers) corresponding to the XerC and XerD binding sites were designated in this study as *dif ^XerC^* and *dif ^XerD^*, respectively, whereas the central hexanucleotide between the two Xer binding sites was named as *dif ^cent^* (Figure 1A). Analysis of the consensus revealed that the *dif ^XerD^* site is better conserved than the *dif ^XerC^* site and that within both *dif ^XerC/D^* boxes, the most conserved region is located in the inner part, near the central region. Regarding *dif ^XerD^*, the adenine residue at position 25 of the 28-nt *dif* sequence is highly conserved, whereas the nucleotides at positions 23 and 24 are more variable (Figure 1A). Within the less conserved nucleotides in *dif ^cent^*, the residue at position 13 (i.e. the second in the hexanucleotide) is the most variable, compared with the other five. Furthermore, the degree of variability upstream and downstream of the 28-mer consensus sequence is high, indicating that the *dif*-related sequences are located in different genetic environments (Figure 1A).

**Figure 2:**
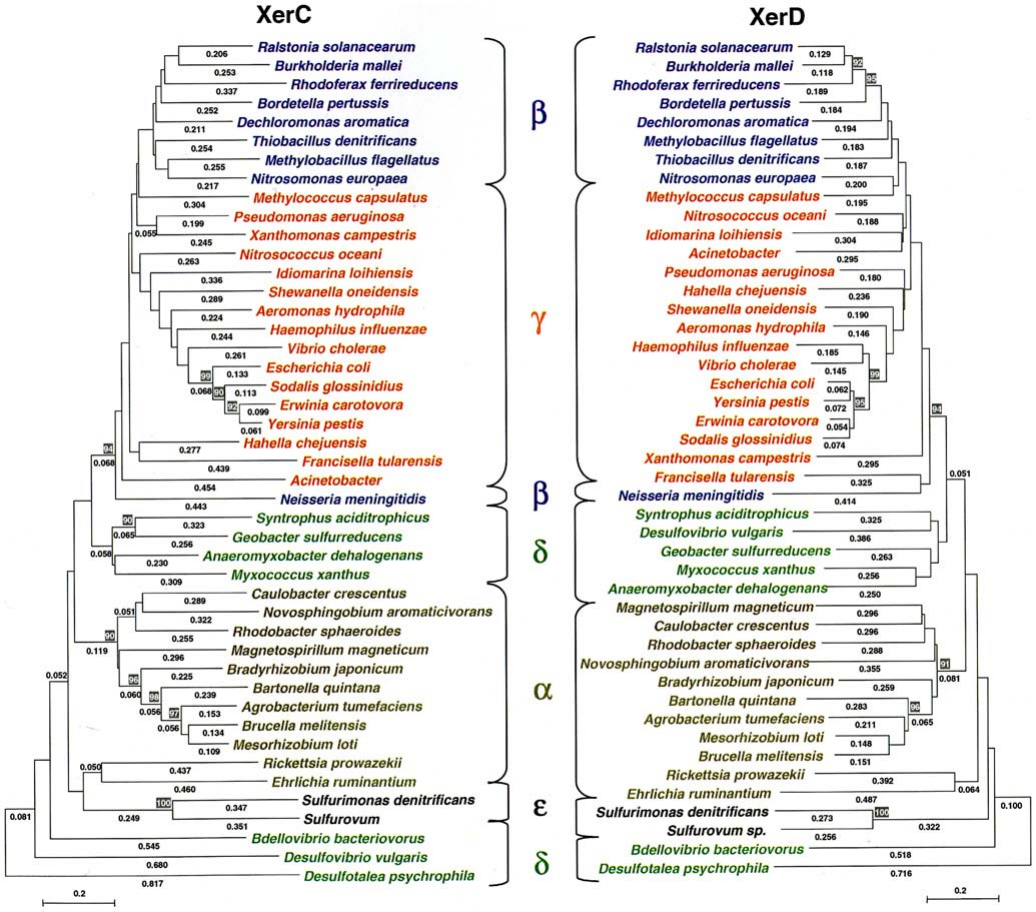
Phylogeny of proteobacterial XerC and XerD recombinases. Representative proteobacterial species of each taxon were selected for the analysis (Table 1). β-proteobacterial species are represented in blue, with γ in red, δ in green, α in magenta and ε in black. Amino acid sequence alignments were performed using Clustal W (MEGA 4 [60]). The evolutionary history was inferred by using the Neighbor-Joining method [61] conducted in MEGA4. Similar results were obtained using the Minimum Evolution method (data not shown). Only significant bootstrap values (≥90%) obtained with 1000 runs are indicated next to the branches (white with a grey background). The tree is drawn to scale, with branch lengths (below the branches) in the same units as those of the evolutionary distances used to infer the phylogenetic tree. Branch lengths below the value 0.05 are not shown. The evolutionary distances were computed using the Poisson correction method and are given as the number of amino acid substitutions per site.

Since *dif ^XerC^* is more variable than *dif ^XerD^*, we then wondered whether XerC recombinases would be less well conserved than the XerD proteins. To answer this question, a phylogenic analysis based on the amino acid sequences was performed on both recombinases in the 48 species which were held to be representative of the proteobacterial taxa (Table 1 and Figure 2). Firstly, our analysis revealed a clustering of the Xer recombinases that confirms the taxonomic organization proposed by Olsen et al [37] (i.e. clustering of the γ and β groups on one hand and the δ, ε and α groups on the other). Secondly, comparison of the XerC and XerD phylogenic trees revealed greater branch lengths in XerC's phylogeny than in XerD's (Figure 2). This clearly indicates greater divergence between the XerC recombinases than between the XerD proteins. The higher variability of the proteobacterial XerC recombinases might thus explain the higher degree of sequence variability for the *dif ^XerC^* site. This observation strongly suggests co-evolution of the Xer recombinases and their related-*dif* sequences. The greater degree of conservation of XerD relative to XerC might be constrained by the direct interaction of XerD (but not XerC) with the highly conserved translocase FtsK [38]. Thus, evolutionary changes in XerD and consequently in *dif ^XerD^*, might have been limited by the conservation of FtsK.

### Intra-species variations in *dif*-related sequences

Although the *dif*-related sequences are highly conserved within a given species, differences in the *dif* sequences were observed between strains. To evaluate any intra-species variations, we compared the *dif*-related sequences in the 21 multi-strain *dif* ^+^ species (Table S1) and calculated the degree of variability at each nucleotide position in the *dif* locus (Figure 1B). This analysis again revealed that the *dif ^XerD^* site is best conserved and that intra-species differences are located at the *dif ^XerC^* and *dif ^XerD^* outer ends (nucleotides 1 to 4 and 27–28, respectively). Surprisingly, with regard to the high nucleotide variability of the *dif ^cent^* in the consensus sequence (Figure 1A), this region displays low intra-species variability. This observation clearly indicates that *dif ^cent^* is well conserved within strains of the same species but weakly conserved between species.

### Variations in *dif*-related sequences in multi-chromosome bacteria

In α, β and γ-proteobacteria, some species contain two or three chromosomes, with each (except in *Agrobacterium tumefaciens*) displaying one *dif*-related sequence (Table S3). Comparison of the sequences in given species indicated that (i) each chromosome harbors a distinct *dif* sequence, (ii) the main differences were found in the *dif ^cent^* region and (iii) the *dif ^xerD^* region was less variable than the *dif ^xerC^* region (Figure 1B). Interestingly, the genes encoding the XerC and XerD tyrosine recombinases were always found as single copies, within the largest chromosome (Table S3) indicating that one couple of Xer recombinases interacts with two (or even three) distinct *dif* sequences in multi-chromosome bacteria and confirming the recent report by Val et al.[16]. This observation suggests that the recombinase / *dif* interaction allows some degree of variability - especially for XerC / *dif* ^XerC^. It is noteworthy that nucleotide positions 5 and 8 to 11 in *dif ^xerC^* and 18 to 23 in *dif ^xerD^* do not vary between chromosomes (Figure 1B) and therefore these positions may well be critical for recombinase binding.

When scanning the genome of the multi-chromosome *A. tumefaciens* for *dif*-related sequences, we found a *dif* sequence on the larger circular chromosome (2.84 Mb) but none on the smaller linear chromosome (2.07 Mb). This finding is not surprising, as it has been shown that the *E. coli dif* sequence is dispensable after linearization of the circular chromosome [39]. The origin of the linear chromosome in *A. tumefaciens* is unknown but some sequence features suggest that it derives from a plasmid [40], [41]. If the plasmid origin of the linear chromosome is confirmed, one can hypothesize that the *dif* sequence would have been lost after the chromosome became linear. Furthermore, when analyzing the presence of Xer homologs in *A. tumefaciens*, we observed that the gene coding for the XerD-like recombinase is present on the linear chromosome, whereas the *xerC* homolog gene was located on the circular chromosome. This distribution of the Xer recombinase genes seems to be specific to *A. tumefaciens*, since both recombinases are located on the larger chromosome in the recently sequenced genomes of *A. vitis* and *A. radiobacter*. Hence, *A. tumefaciens* is the only known multi-chromosome bacterium in which the XerC and XerD-encoding genes are on different chromosomes. This example suggests a *xer* gene translocation from the larger, conserved chromosome to the smaller, less conserved one [42].

### Low G+C content, palindromicity, close association with the terminus and presence in a non-coding region are conserved features of the *dif*-related sequences

Nucleotide analysis of the *dif*-related sequences revealed that for a given species, the G+C content of the *dif* motif was systematically lower than the G+C content of the corresponding chromosomes (a difference of between 8.3% and 58.4%, median value  = 29.9%) (Table S1). Furthermore, as palindromicity seems to be essential for *dif* functionality [11], [43], we next searched for palindromes in the *dif* sequences from the 48 selected species. Of the 28 nucleotides of the *dif* sequence, 16.3±3.0 are involved in a palindrome. When the analysis was performed with 28-mers randomly generated from the initial *dif* sequences, the number of nucleotides involved in palindrome was significantly lower (9.3±4.4) (p<0.001; Student's test, n = 48) confirming that palindromicity is a key feature of the *dif* motif. We then analyzed each nucleotide position in the *dif* sequences for their involvement in a palindrome. Positions 5, 6, 23, 24 of the 28-mer are rarely involved in a palindromic structure, whereas nucleotides at position 8 to 11 and 18 to 21 (corresponding to the inner part of *dif ^XerC^* and *dif ^XerD^*) are frequently associated (Figure S1).

**Figure 3:**
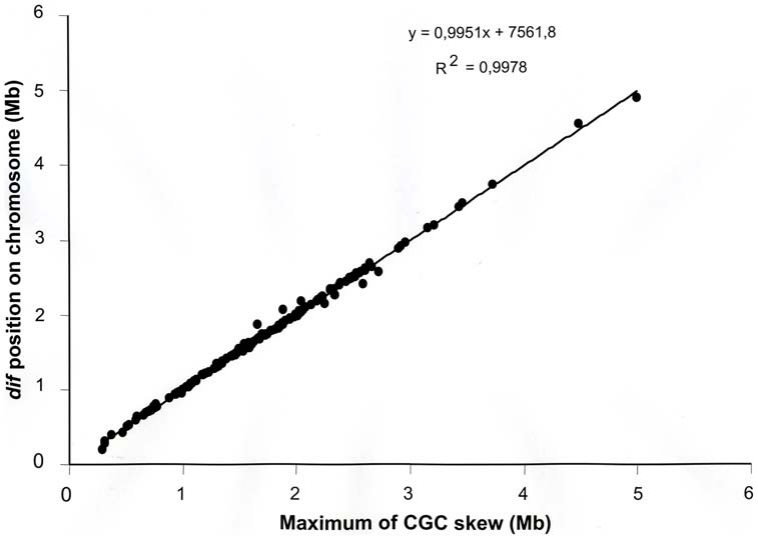
Correlation between the position of the *dif* sequence and the terminus of replication as defined by cumulative GC skew. The analysis was performed on the 161 proteobacterial chromosomes from the 137 representative *dif* ^+^ species (Table S1). Chromosome of *Wolbachia* endosymbiont of *Drosophila melanogaster* and chromosome 2 of *Pseudoalteromonas haloplanktis* were not included in the analysis since no terminus of replication could be located for these species by the method of the cumulative GC skew. The equation of the plot and the coefficient of determination (R^2^) are given.

We then compared the *dif* position on the chromosome relative to the maximum cumulative GC skew. On the 161 chromosomal sequences, the median distance between the *dif* motif and the replication terminus as defined by the cumulative GC skew method was calculated to be 7277 bp (first quartile = 2055.5; third quartile = 21,445.5), with a distance ranging from 146 bp for chromosome 1 in *V. cholerae* to 199612 bp for *Syntrophus aciditrophicus*. The great distance between the location of *dif* and the peak of the cumulative GC skew curve for a few species can be mainly explained by a noisy GC skew signal blurring the precise location of its maximum value. However, despite this difficulty, we noted a high degree of correlation (R^2^ = 0.9978) between *dif*'s position and the peak of the cumulative GC skew curve (Figure 3), which confirms the close association previously observed in a smaller number of species [22].

Furthermore, we analyzed the gene environment of the 161 *dif* motifs and found that most (96.3%) were located in non-coding regions. This observation clearly indicates that *dif* intergenicity is another key feature. However, in a few cases (6 sequences out of 161, 3.7%), *dif* was present within coding sequences, four of which corresponding to hypothetical proteins, plus two associated to characterized open reading frames (ORFs). Whereas the sequence was inserted within a bacteriophage protein-coding sequence in *Vibrio parahaemolyticus* (chromosome 1), the motif was located in a gene coding for a major facilitator family transporter in the third chromosome of *Burkholderia ambifaria* (Table S1). Analysis of the flanking coding sequences of the 161 representative *dif*-related sequences revealed that 10.9% were flanked by proteins of phage origin and 14.2% were associated with insertion sequences or transposase- or integrase-encoding genes. This shows that about a quarter of the *dif* sequences are associated with ORFs whose products are involved in mobility. This number might even be an underestimate, since 60% of the *dif* sequences have ORFs with unknown functions in their vicinity (upstream, downstream or both). These results emphasize the propensity of the terminus region in general and *dif* in particular to facilitate DNA mobility.

### Two *dif*/Xer systems in ε-proteobacteria

**Figure 4:**
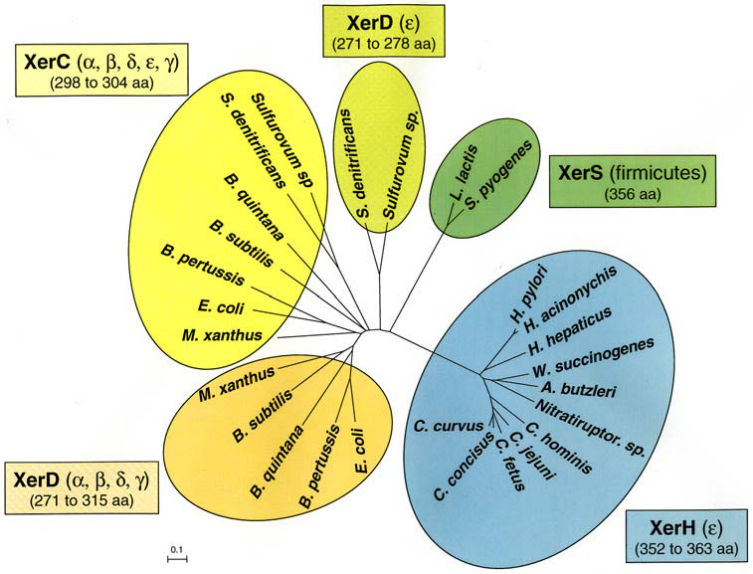
Phylogenetic analysis of XerC, XerD, XerH and XerS recombinases. XerH from the ε subgroup species (listed in Table 2) were compared with XerD and XerC recombinases from other ε species and representative bacteria from the α, β, δ and γ taxa (Table 1). XerS recombinases of *S. pyogenes* M1 GAS and *L. lactis* Il1403 [23] were added for comparison. Amino acid sequence alignment (with Clustal W) and phylogenetic analyses were performed in MEGA4 [60]. The phylogeny was built using the Neighbor-Joining method [61]. The tree is drawn to scale, with branch lengths in the same units as those of the evolutionary distances used to infer the phylogenetic tree. The evolutionary distances were computed using the Poisson correction method and are in the units of the number of amino acid substitutions per site. The size range of the recombinases (in amino acids) is indicated under the recombinase name, in brackets.

**Figure 5:**
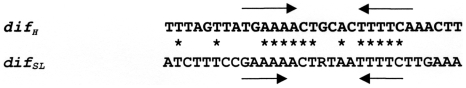
Alignment of *dif_H_* and *dif_SL_*. The *dif_H_* sequence corresponds to the putative *dif* motif of *H. pylori* 26695 (Table 2), whereas *dif_SL_* was described by Le Bourgeois et al. [23]. Asterisks indicate the common nucleotides and arrows designate inverted repeats.

During the initial analysis of the ε-proteobacterial chromosomes, we found that only one species (*Sulfurimonas denitrificans*) out of 8 had a *dif* sequence (Table 1 and Table S1). In order to understand the apparent absence of a *dif*-related sequence in the genome of *Helicobacter* and *Campylobacter* species, we searched for the presence of the Xer-like recombinases in this subgroup. A XerD homolog was found in all bacteria belonging to this ε-subgroup, although the corresponding protein had a low degree of homology with *E. coli* XerD and was longer (between 353 and 363 amino acids versus 298 amino acids for *E. coli* XerD). Surprisingly, we did not detect any other recombinases that unambiguously corresponded to a XerC homolog. Blastp analysis with *E. coli* XerC showed the presence of XerC-like recombinases but none was ubiquitously found in the *Helicobacter* and *Campylobacter* species. Some of these XerC-like recombinases probably correspond to the transposable element-associated recombinases found in *Helicobacter* and designated “XerT” by Kersulyte et al. [44]. We thus concluded that this ε-proteobacteria sub-group expresses only one ubiquitous Xer recombinase that we designated here as “XerH” because *Helicobacter* is a major representative of this group. The presence of a single Xer recombinase is not unique in the bacterial kingdom. Indeed, it was recently shown that *Streptococcus* and *Lactococcus* species display an unconventional *dif* sequence (*dif_SL_*) which requires a single 356-amino acid recombinase, XerS [23]. Although XerS and XerH exhibit a similar size, the proteins appear to be phylogenetically unrelated (Figure 4). However, when BLASTing *dif_SL_* on ε-proteobacteria genomes, we discovered a *dif*_SL_ homolog presenting all key features of a *dif* motif, i.e. (i) located near to the peak of the cumulative GC skew, (ii) present in non-coding regions and (iii) with a low G+C content (Table 2). Furthermore, this *dif_SL_*–like sequence designated as *dif_H_* was composed of 2 highly conserved, inverted repeats separated by a central hexanucleotide variable region - another hallmark of *dif* (Figure 5). A *dif_H_* sequence was also found in chromosomes of ε-proteobacteria sequenced after January 1^st^, 2007 (*Arcobacter butzleri*, *C. concisus*, *C. hominis*, *C. doylei* and *Nitratiruptor sp*) (Table 2). It is noticeable that in most ε-species, *dif_E_* is genetically linked to the recombinase-encoding gene, the pair corresponding to an individual genetic module, as defined by Le Bourgeois et al. for *dif_SL_* and *xerS* (Table 2) [23]. Interestingly, the recently sequenced ε-species *Sulfurovum sp* did not have a *dif_H_* sequence but did possess a more classical motif with homology to the *dif* sequence of the taxonomically-related *Sulfurimonas denitrificans*. Hence, two distinct groups can now be defined in the ε taxon as a function of their *dif*/Xer system. One encompasses most of the epsilon species (*Campylobacter* sps, *Helicobacter* sps, *Wolinella succinogenes*, *Arcobacter butzleri* and *Nitratiruptor*) with *dif_H_* sequences similar to the firmicutes' *dif_SL_* and which, in most species, is genetically linked with *xerH*, a single Xer recombinase-encoding gene. Another group (*Sulfurimonas denitrificans* and *Sulfurovum*) displays the classical features of the *dif*/Xer system i.e. a *dif* sequence with homology to the canonical *dif* and two recombinases genes scattered across the chromosome. Interestingly, the two groups belong to two distinct clades [45], suggesting that the *dif*/Xer recombination systems are associated with specific phylogenic groups. Experimental approaches are now required to test the functionality of the newly discovered ε Xer-like recombination system.

**Table 2:** Features of the putative *dif* sequences of ε-proteobacteria.

ε-proteobacteria species (1)	chromosomal features (2)			putative *dif* characteristics					
	size (bp)	G+C content	CGC skew	sequence (40-mer) (3)	G+C content	position on chromosome	distance from GC skew (bp)	distance from *xerE* (bp)	intergenic location (4)
				***dif_H_***					
*Arcobacter butzleri* RM4018	2341251	0.270	1232417	TTAATTAGTATTGAAAACT**A**TAATTTTCAAATAAAATATA	0.100	1197716	34701	66	yes
*Campylobacter concisus* 13826	2052006	0.394	971215	ATATTTTGTATTGAAAACT**A**TAATTTTCAAATTGATATTT	0.125	999842	28627	59459	hyp. prot
*Campylobacter curvus* 525.92	1971264	0.445	1008113	ATATTTTGTATTGAAAACT**A**TAATTTTCAAATTAATATTT	0.100	991768	16345	68030	hyp. prot
*Campylobacter fetus subsp. fetus* 82-40	1773615	0.333	908630	TTATTTTGTATTGAAAACT**A**TAATTTTCAAACTATTATGA	0.150	886842	21788	35968	yes
*Campylobacter hominis* ATCC BAA-381	1711272	0.317	875024	TATTTTATTTTTGAAAACT**A**TAATTTTCAAACTTTTTTGT	0.125	851021	24003	214673	yes
*Campylobacter jejuni subsp. doylei* 269.97	1845106	0.306	853771	TAATTTTGTATTGAAAACT**A**TAATTTTCAAACTTTTTTAT	0.125	892865	39094	217	yes
*Campylobacter jejuni* RM1221	1777831	0.303	893799	TAATTTTGTATTGAAAACT**G**TAATTTTCAAACTTTTTTAT	0.150	888360	5439	215	yes
*Helicobacter acinonychis str. Sheeba*	1553927	0.382	748099	TAGTTTAGTTATGAAAACT**G**CACTTTTCAAACTTTTAAAT	0.225	747275	824	282	yes
*Helicobacter hepaticus* ATCC 51449	1799146	0.359	1794500 (5)	TGAATTAGTTATGAAAACT**A**TACTTTTCAAACTTTTTTAT	0.175	1765790	28710	125	yes
*Helicobacter pylori* 26695	1667867	0.388	813426	TCATTTAGTTATGAAAACT**G**CACTTTTCAAACTTTTAAAT	0.225	723517	89909	1981	yes
*Nitratiruptor sp*. SB155-2	1877931	0.397	927614	TTTATTAGTATTGAAAACT**A**TAATTTTCAAACTTTTATTT	0.125	1001399	73785	52	yes
*Wolinella succinogenes* DSM 1740	2110355	0.484	1188027	TCATTTAGTATTGAAAACC**A**TAATTTTCAAACTCATAATT	0.200	1170384	17643	16	yes
consensus sequence (6)				----TT--T--TGAAAAC---A-TTTTCAAA---------					
				***classical dif***				distance from XerC / XerD (bp)	
*Sulfurimonas denitrificans* DSM 1251 (7)	2201561	0.345	1135161	AAATACTTTCAATAGAATT**T**ACATTATGTTAACCAATATA	0.175	1122264	12897	705981/193695	yes
*Sulfurovum sp*. NBC37-1	2562277	0.439	1189307	TTGCTTTTTTAATAGAATT**T**ATATTATGTTAATCAATAGA	0.150	1186929	2378	1109695/758085	yes
consensus sequence (6)				------TTT-AATAGAATTTA-ATTATGTTAA-CAATA-A					

(1) One representative per species.

(2) All genomes are circular.

(3) The position of the putative *dif* motif on the chromosome corresponds to the nucleotide in bold type, located between the two inverted repeats.

(4) hyp. prot.  =  hypothetical protein.

(5) maximum of the GC skew.

(6) Underlined nucleotides correspond to the inverted repeats.

(7) Sulfurimonas denitrificans strain DSM 1251  = Thiomicrospira denitrificans ATCC 33889.

### The *dif*/Xer system is not present in all proteobacteria

Our approach revealed that 12.2% of the studied proteobacterial species do not contain a *dif* motif. Most of them lack the XerC, XerD, XerH or XerS recombinases, justifying the absence of *dif* (Table S4). It seems that genome size should be taken into account when considering the absence of the *dif*/Xer system. Indeed, insect endosymbiont bacteria (*Buchnera* sp, *Blochmannia* sp, *Carsonella ruddii*, *Ruthia magnifica*, *Baumannia cicadellinicola and Wigglesworthia glossinidia*) have a genome size ranging from 0.159 to 1.1 Mb and lack the *dif*/Xer system (Table S4). During their co-evolution with their host, the endosymbiotic bacteria have lost a large part of their genome and have retained only genes that are essential for survival [46]. The absence of the *dif*/Xer system in these bacteria indicates that this recombination system is not required for microbial symbiosis. Likewise, the marine α-proteobacteria *Pelagibacter ubique* has the smallest known genome of a free-living microorganism (1.3 Mb) [47] and, like the endosymbiotic bacteria, does not possess a *dif*/Xer system. This confirms that genome fitting can affect non-vital systems, such as the Xer machinery. However, low chromosome size is not always associated with the absence of the *dif*/Xer system, since the Rickettsiales (α-proteobacteria with a genome ranging from 0.85 to 1.52 Mb in size) do harbor *dif*/Xer recombination machinery (Table 1 and Table S1). Furthermore, the absence of the *dif*/Xer system cannot only be explained by chromosome fitting, since bacteria with a larger chromosome (like the Legionellales, *Colwellia psychrerythraea* or *Saccharophagus degradans*: genome size ranging from 2 Mb to 5 Mb) also lack this machinery. Surprisingly, a *dif*/Xer system was not found in *Aromatoleum aromaticum* str EbN1 (also designated as *Azoarcus* sp EbN1) whereas the complete system was revealed in *Azoarcus* BH72. This difference could be attributed to the low degree of synteny seen for the genomes of these two phylogenetically similar species [48].

Chromosome dimerization is a prerequisite for *dif*/Xer activity and requires the presence of RecA, RecBC, and RecF pathways for homologous recombination between sister chromosomes, RecA being the most efficient for this function [49], [4]. Except for *Candidatus Ruthia magnifica* which does not display RecA, RecB or RecF homologs, all other *dif*-deficient species encode at least one enzyme that may be responsible for chromosome dimerization (Table S4). This observation raises the question of the fate of bacterial cells in which dimerization occurs without the rescue by the *dif*/Xer system.

## Discussion

In the present study, 234 chromosomes from 156 proteobacterial species were analyzed for the presence of a *dif*-related sequence by using a strategy mainly based on homology with experimentally-defined *dif* sequences and a close association with the chromosome terminus defined by the cumulative GC skew. We now have an overview of the features of the *dif*/Xer systems present in proteobacteria. Most species display a “classical” *dif* sequence composed of two undecanucleotides, a conserved *dif ^XerD^* and a more variable *dif ^XerC^* separated by an hexanucleotide region (*dif ^cent^*). These *dif* motifs (i) contain inverted repeats forming a palindrome, (ii) are located intergenically, with no apparent specific genetic environment, (iii) have a lower G+C content than the chromosomal G+C content and (iv) are located near the replication terminus as identified in GC skew analyses. These sequences are found in bacteria harboring XerD- and XerC-like recombinases. Other proteobacteria, notably a subgroup of ε-proteobacteria, display a sequence (*dif_H_*) which is homologous to *dif*_SL_ from *streptococci* and *lactococci* [23]. As the canonical *dif* motif, *dif_H_* (i) exhibits a low G+C content (ii) is located intergenically, near the terminus defined by the GC skew, (iii) is not associated with specific genetic elements or open reading frames, (iv) displays a palindromic structure and, (v) like *dif_SL_*, can be located in the immediate vicinity of its recombinase. Furthermore, as for the streptococci and lactococci, a single Xer-like recombinase (XerH) was found in species displaying a *dif_H_* sequence. However, no phylogenic association between XerS and XerH could be found, which strongly suggests the existence of two unrelated *dif*/Xer systems. Taken as a whole, these data demonstrate that at least two types of *dif*/Xer systems exist in proteobacteria: the classical machinery found in most species and an atypical system present in a sub-group of ε proteobacteria. Exhaustive analysis of the *dif*/Xer systems in other bacterial taxa is now required to evaluate the distribution of these systems in the bacterial kingdom. The general features of *dif* defined in our study should facilitate this investigation.

Our analysis also demonstrated that the *dif*/Xer system is not as universal as initially thought. Indeed, 12.2% of the studied proteobacterial species do not harbor this recombination machinery - an absence that could be explained by genome fitting for small genome microorganisms but not for bacteria with large chromosomes (like the Legionellales, *Saccharophagus degradans* or *Colwellia psychrerythraea*). It is presently unclear whether the large chromosome in these microorganisms lost the Xer recombination system, never acquired it or developed a substitutive system to deconcatenate the chromosomes. The consequences of this absence are also intriguing, as most of these *dif*-deficient species seem to possess the enzymatic machinery (RecA, RecBCD and RecF) potentially responsible for chromosome dimerization by homologous recombination (Table S4) [49], [4]. In the absence of *dif* and Xer recombinase, how do bacterial cells handle chromosome deconcatenation? Can these bacteria survive without the need to resolve chromosome dimers or does an alternative recombination system replace the *dif*/Xer system? It has already been shown that the *loxP*/Cre resolvase system (but not *res*/Tn3) can suppress the filamentation phenotype of a *dif*-deficient *E. coli* but only when *loxP* is located at the chromosome terminus [6]. This demonstrates that the *dif*/Xer machinery can be replaced by other recombination systems. However, there is presently no evidence to suggest that *dif*/Xer-deficient proteobacteria harbor *loxP*/Cre resolvase-like systems. In the case of *Legionella*, the absence of *dif*/Xer agrees with an early observation showing filamentous cells in *Legionella* cultures [50]. Experimental evidence is now required in order to establish whether the filamentous phenotype in *L. pneumophila* results from the absence of *dif*/Xer recombination. This question could be answered by reintroducing a functional *dif*/Xer system into *Legionella* and then checking for the filamentous phenotype.

Compared with other recombination targets, the *dif* motif harbors a particular structure in view of the presence of two recombinases. It is composed of two recombinase–specific outer regions and two inner regions with dyad symmetry, close to the central hexanucleotide. Our analysis of nucleotide variability in proteobacteria species revealed that the inner regions of *dif ^XerC^* and *dif ^XerD^* are highly conserved, whereas the outer regions are much more variable (Figure 1A). Nucleotides at position 23 and 24 (located in the outer part of *dif ^XerD^*) are highly variable and are rarely part of the palindrome. Interestingly, these positions were experimentally defined in *E. coli* as major contributors to the XerD binding specificity [43] and analysis of the crystal structure of XerD predicted that the *dif* nucleotide at position 24 interacts directly with the highly conserved amino acid residue Q221 of XerD [51]. Furthermore, this position is much less variable in multi-strain species and multichromosome species (Figure 1B). This observation shows that the variability of the nucleotide at position 24 is primarily inter-species variability and could even be considered as a species marker. Lastly, an adenine residue is highly conserved at position 25 within the outer part of *dif ^XerD^* and could represent a general feature of the *dif* in proteobacteria.

As for *dif ^XerD^*, the variable outer region of *dif ^XerC^* corresponds to the recombinase binding site, since positions 2 and 5 have been described as major contributors to XerC binding [43]. For the outer *dif ^XerC^* region, our study shows that nucleotides in position 2 of *dif* display the highest variability, whereas the residue located in position 5 is the least variable. It would be interesting to know whether the most conserved position in *dif ^XerC^* (position 5) is associated with a conserved amino acid residue in XerC. Unfortunately, structure/function analysis of XerC is prevented by the lack of structural data.

The *dif ^cent^* was a hexamer in all the proteobacterial genomes that we analyzed, suggesting that the size of the central region separating the recombinase binding sites is a critical feature. In *E.coli*, it has been demonstrated that the 6 bp-distance between the XerC and XerD binding sites was optimal for chromosomal recombination activity and cleavage [52], [53]. A 8-bp central region is found in natural plasmids like ColE1 but is always associated with adjacent DNA sequence and accessory proteins [54], [55], [56]. The presence of a 6-bp central region in proteobacteria thus suggests that chromosomal recombination at *dif* in these species does not require accessory elements. Furthermore, positions in the central hexamer do not appear to be equivalent. Indeed, our overall analysis suggests that the nucleotide at position 13 within *dif ^cent^* is highly variable (Figure 1A), whereas it is the least variable residue of the hexanucleotide in the genomes of multi-chromosome bacteria (Figure 1B). Hence, this position may represent an important feature for species discrimination. Moreover, in multi-chromosome species, *dif ^cent^* is more variable than the *dif ^XerC^* or *dif ^XerD^* regions (Figure 1B). This observation agrees with the study by Val et al. [16] and confirms that the central hexanucleotide is a key region for discriminating between chromosomes within the same bacterium and for avoiding chromosome fusion.

This study represents the first comprehensive analysis of the *dif* motif and its recombinases; it revealed a new *dif*/Xer recombination system in proteobacteria and constitutes an important step toward the characterization of the *dif*/Xer-like systems in bacteria with circular chromosomes.

## Methods

### Identification of *dif*-like motifs in proteobacteria

The *dif*-related sequences were identified by using genomic similarity search tools, such as the Basic Local Alignment Search Tool (BLAST) (http://www.ncbi.nlm.nih.gov/sutils/genom_table.cgi) [57] and the YASS DNA pairwise alignment tool (http://bioinfo.lifl.fr/yass/yass.php) [58]. Sequences of the experimentally characterized *dif* elements from *E. coli, B. subtilis, C. crescentus, X. campestris*, *V. cholerae*, streptococci and lactococci [13], [14], [15], [16], [23] were used as query sequences. Given that previous studies had revealed conservation of the *dif* sequence, we used this feature to develop an approach for characterizing *dif* homologs in phylogenetically related species.

Our analysis of *dif*-related motifs was performed on all the 234 completed proteobacterial chromosome sequences released before January 1^st^, 2007 (Table S1). This corresponds to 156 species and represents 53.1% (234 out of 440) of all the bacterial chromosomes sequenced as of that date. The nucleotide sequences were downloaded from the National Center for Biotechnology Information (NCBI, http://www.ncbi.nlm.nih.gov). Forty-eight species were selected as being representative of the different proteobacterial taxa (Table 1). Information on the coding sequences flanking the *dif*-related sequence was obtained from the protein tables (.ptt file) summarizing the genome annotation at NCBI.

### Skew analysis

The position of the candidate *dif* sequences was compared with that of the DNA replication terminus, as defined by the maximum of cumulative GC nucleotide (CGC) skew obtained by nucleotide skew analyses of the chromosome sequences [59]. When the maximum of CGC skew was undetectable, we chose the maximum of GC skew (GC) of the chromosome or at the first position of the codon (GC1).

### Determination of the *dif* consensus sequence

To define the *dif* consensus sequence for proteobacteria, we aligned the *dif*-related sequences extracted from the available chromosomes. To avoid redundancy when several genome sequences were available for one bacterial species, only the information on the first-published chromosome (according to the NCBI release date) was used. In the end, 161 bacterial chromosomes were selected for determination of the *dif* consensus sequence; since some species have several chromosomes, the number of chromosomes is higher than the number of species. The degree of nucleotide variability (*v*) at each position of the 28-mer was defined as *v* = 1–*f*, where *f* is the frequency of the most frequent nucleotide.

### Measurement of the degree of variability

The intra-species nucleotide variability of the *dif* sequence was measured in the 21 *dif* ^+^species represented by at least two strains (termed “multi-strain species” in this study). Intra-species variability was measured at each position of the 28-nucleotide *dif* sequence. A score of 1 was attributed to the position when the nucleotides differed between strains of the same species; if not, the score at this position was 0 (i.e. conservation). For each position of the 28-nucleotide *dif* sequence, the scores obtained for all the species were added and normalized against the number of species (n = 21). This value obtained corresponds to the degree of variability at each position and, hence, a low value corresponds to low nucleotide variability at the position.

A similar approach was adopted for analyzing the nucleotide variability of *dif* in 19 out of 20 multi-chromosome species (listed in Table S3). Multi-chromosome *Agrobacterium tumefaciens* was not included in the analysis since only one of its two chromosomes display a *dif* sequence. Within the same strain, chromosomes were compared in terms of *dif* sequence. A score of 1 was attributed to the position if the nucleotides differed for the 2 or 3 chromosomes in the same strain; if not, the position was scored as 0. Next, for each position, the scores were added and normalized against the number of species (n = 19) to obtain a value representing the degree of variability in multi-chromosome species, a low value being associated with a low nucleotide variability at the position.

### Palindromicity

Palindromicity was analyzed by comparing the 28-nt *dif* sequence with its inverted complementary counterpart in the 48 selected proteobacterial species (Table 1). The palindrome was defined as the conserved nucleotide sequence between *dif* and its inverted, complementary strand. When a nucleotide was found both in *dif* and in the reverse complementary sequence, a value of 1 was given to the position. Next, the values for the 48 *dif* sequences for each position were added together to give the *n* value. The palindromicity frequency (*fpal*) was then estimated as: *fpal* = *n*/48, with 48 being the number of *dif* sequences analyzed. A *fpal* value of 1 to a nucleotide position means that the nucleotide is always part of a palindrome.

In order to demonstrate that the presence of a palindrome is a key feature of *dif* motifs, we compared each *dif* sequence with a randomly generated 28-mer obtained by shuffling the nucleotide of the original *dif*. Next, the nucleotides involved in the palindrome were counted in both *dif* and the randomized 28-mers and the average numbers of nucleotide involved in a palindrome were calculated and compared.

### BLASTp analysis and the phylogeny of the Xer recombinases

BLASTp analysis were performed using reference amino acid sequences from *E.coli* K12 XerC and XerD recombinases (protein reference on NCBI: NP_418256 and NP_417370, respectively), from *Lactococcus lactis* Il1403 XerS (NP_267388) and from *E. coli* K12 RecA, RecB and RecF (NP_417179, NP_417297.1 and NP_418155.1, respectively).

Phylogenetic analysis of the Xer recombinases was performed with MEGA version 4 [60]. Sequences were aligned with ClustalW, whereas phylogeny was build using the Neighbor-Joining method [61].

## Supporting Information

Figure S1 Palindromicity of the dif-related sequences. The frequency of palindromicity was calculated from the 48 representative dif sequences (Table 1), as described in the Methods section. Black bars represent dif XerC and dif XerD nucleotides, whereas grey bars correspond to dif cent nucleotides. White bars represent nucleotides outside dif.
(6.24 MB TIF)

Table S1 Genome, dif, and Xer recombinase features of 234 protobacteria
(0.17 MB XLS)

Table S2 Nucleotide frequency (%) of the dif-related sequences from 161 proteobacterial chromosomes.
(0.07 MB DOC)

Table S3 dif-related sequences in multi-chromosome proteobacteria
(0.15 MB DOC)

Table S4 Xer recombinases and RecA, RecB and RecF homologs in dif-deficient proteobacteria
(0.03 MB XLS)
