# Supplementary figures and images for: The dif/Xer Recombination Systems in Proteobacteria
Source: PLoS One. 2009 Sep 3;4(9):e6531. doi: 10.1371/journal.pone.0006531 (PMC2731167; doi:10.1371/journal.pone.0006531)

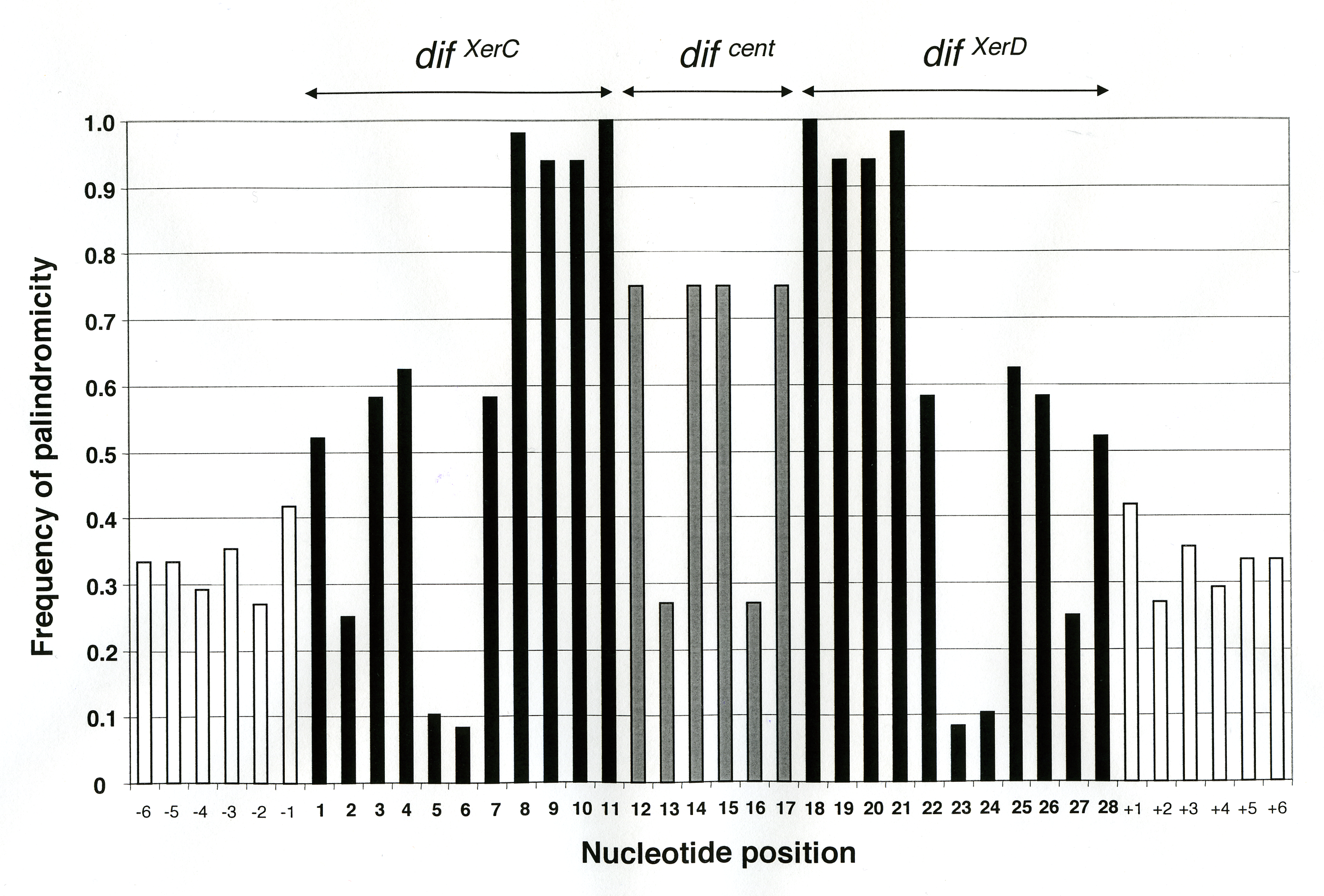

Supplement: Figure S1 — Palindromicity of the dif-related sequences. The frequency of palindromicity was calculated from the 48 representative dif sequences (Table 1), as described in the Methods section. Black bars represent dif XerC and dif XerD nucleotides, whereas grey bars correspond to dif cent nucleotides. White bars represent nucleotides outside dif. (6.24 MB TIF) [file pone.0006531.s001.tif]
