# Supplementary material for: The dif/Xer Recombination Systems in Proteobacteria
Source: PLoS One. 2009 Sep 3;4(9):e6531. doi: 10.1371/journal.pone.0006531 (PMC2731167; doi:10.1371/journal.pone.0006531)
Supplement: Table S2 — Nucleotide frequency (%) of the dif-related sequences from 161 proteobacterial chromosomes. (0.07 MB DOC) [file pone.0006531.s003.doc]

**Table S2: Nucleotide frequency (%) of the *dif*-related sequences from 161 proteobacterial chromosomes.**

|  | nucleotide position ^(1)^ | | | | | | | | | | | | | | | | | | | | | | | | | | | | | | | | | | | | | | | |
| --- | --- | --- | --- | --- | --- | --- | --- | --- | --- | --- | --- | --- | --- | --- | --- | --- | --- | --- | --- | --- | --- | --- | --- | --- | --- | --- | --- | --- | --- | --- | --- | --- | --- | --- | --- | --- | --- | --- | --- | --- |
|  | **-6** | **-5** | **-4** | **-3** | **-2** | **-1** | **1** | **2** | **3** | **4** | **5** | **6** | **7** | **8** | **9** | **10** | **11** | **12** | **13** | **14** | **15** | **16** | **17** | **18** | **19** | **20** | **21** | **22** | **23** | **24** | **25** | **26** | **27** | **28** | **+1** | **+2** | **+3** | **+4** | **+5** | **+6** |
| **A** | **39,8** | **37,9** | 28,0 | 27,3 | **38,5** | **39,8** | **52,8** | **34,8** | 18,0 | 12,4 | 1,2 | 13,7 | 1,2 | **99,4** | 0,0 | **91,3** | **99** | 1,9 | 31,7 | 8,7 | **77,6** | 4,3 | **84,5** | 0 | 0 | **100** | 0 | 0,0 | 1,2 | 34,8 | **98,8** | **83,2** | **75,2** | 5,0 | **49,7** | **44,1** | **43,5** | **40,4** | 24,2 | **39,8** |
| **T** | 34,8 | 28,6 | 23,6 | 28,0 | 37,3 | 37,9 | 25,5 | 24,8 | **59,6** | **50,3** | 22,4 | 11,2 | 14,3 | 0,0 | **94,4** | 6,8 | 1 | **82,0** | 16,8 | **83,2** | 6,8 | **67,7** | 1,2 | **100** | **100** | 0 | **100** | 0,0 | **74,5** | **48,4** | 1,2 | 5,6 | 8,7 | **87,6** | 32,9 | 18,0 | 24,2 | 13,7 | **40,4** | 24,2 |
| **G** | 10,6 | 8,7 | 8,1 | 13,0 | 16,1 | 9,9 | 15,5 | 25,5 | 17,4 | 34,8 | 4,3 | **52,2** | 23,0 | 0,6 | 0,6 | 1,2 | 0 | 13,7 | **43,5** | 4,3 | 14,9 | 11,8 | 8,7 | 0 | 0 | 0 | 0 | **100** | 19,3 | 5,0 | 0,0 | 0,6 | 6,8 | 3,1 | 6,2 | 23,6 | 21,1 | 26,1 | 23,0 | 19,9 |
| **C** | 14,9 | 24,8 | **40,4** | **31,7** | 8,1 | 12,4 | 6,2 | 14,9 | 5,0 | 2,5 | **72,0** | 23,0 | **61,5** | 0,0 | 5,0 | 0,6 | 0 | 2,5 | 8,1 | 3,7 | 0,6 | 16,1 | 5,6 | 0 | 0 | 0 | 0 | 0,0 | 5,0 | 11,8 | 0,0 | 10,6 | 9,3 | 4,3 | 11,2 | 14,3 | 11,2 | 19,9 | 12,4 | 16,1 |
| (2) | **a** | **a** | **c** | **c** | **a** | **a** | **A** | **a** | **T** | **T** | **C** | **G** | **C** | **A** | **T** | **A** | **A** | **T** | **g** | **T** | **A** | **T** | **A** | **T** | **T** | **A** | **T** | **G** | **T** | **t** | **A** | **A** | **A** | **T** | **a** | **a** | **a** | **a** | **t** | **a** |

^(1)^ Residues from -6 to -1 and from +1 to +6 represent the nucleotides upstream and downstream of *dif*, respectively.

^(2)^ Consensus sequence. When nucleotide frequency at a given position is > 50%, the nucleotide is written in upper case letters otherwise it is represented by lower case letters.
