# Supplementary material for: The dif/Xer Recombination Systems in Proteobacteria
Source: PLoS One. 2009 Sep 3;4(9):e6531. doi: 10.1371/journal.pone.0006531 (PMC2731167; doi:10.1371/journal.pone.0006531)
Supplement: Table S3 — dif-related sequences in multi-chromosome proteobacteria (0.15 MB DOC) [file pone.0006531.s004.doc]

**Table S3: *dif*-related sequences in multi-chromosome proteobacteria**.

|  |  |  |  |  |  |  |
| --- | --- | --- | --- | --- | --- | --- |
|  | chromosome | chromosome | *dif* | *dif*-related sequences ^(1)^ | *xerC* | *xerD* |
| 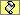Species |  | size (bp) | position |  | homolog | homolog |
|  |  |  |  |  |  |  |
| **α-proteobacteria** |  |  |  |  |  |  |
|  |  |  |  |  |  |  |
| *Agrobacterium tumefaciens* str. C58 | [circular](http://www.ncbi.nlm.nih.gov/entrez/query.fcgi?cmd=Retrieve&db=Nucleotide&list_uids=15887359&dopt=GenBank) | 2841580 | 1478815 | TAATCGCATAA GATATA TTATGGAACTT | **+** | **-** |
|  | linear | 2075577 |  | **No dif** | **-** | **+** |
| *Brucella abortus* biovar 1 str. 9-941 | 1 | 2124241 | 1051563 | **T**AATC**GC**ATAA **G**AT**AG**A TTATGGAAC**TG** | **+** | **+** |
|  | 2 | 1162204 | 682521 | **A**AATC**AG**ATAA **T**AT**GT**A TTATGGAAC**AT** | **-** | **-** |
| *Brucella melitensis* 16M ^(2)^ | 1 | 2117136 | 954739 | **T**AATC**GC**ATAA **G**AT**AG**A TTATGGAAC**TG** | **+** | **+** |
|  | 2 | 1177785 | 758184 | **A**AATC**AG**ATAA **T**AT**GT**A TTATGGAAC**AT** | **-** | **-** |
| *Brucella suis* 1330 | 1 | 2107794 | 1034393 | **T**AATC**GC**ATAA **G**AT**AG**A TTATGGAAC**TG** | **+** | **+** |
|  | 2 | 1207381 | 531784 | **A**AATC**AG**ATAA **T**AT**GT**A TTATGGAAC**AT** | **-** | **-** |
| *Paracoccus denitrificans* PD1222 | 1 | 2852282 | 1343401 | **TTA**ACCGATAA **TGATAG** TTATGTAA**ACC** | **+** | **+** |
|  | 2 | 1730097 | 1170299 | **AAT**ACCGATAA **CCGGGA** TTATGTAA**TAT** | **-** | **-** |
| *Rhodobacter sphaeroides* 2.4.1 | 1 | 3188599 | 1436844 | **GAG**TC**G**GATAA **T**C**TGT**A TTATGTA**T**TC**T** | **+** | **+** |
|  | 2 | 943016 | 371574 | **TTA**TC**T**GATAA **G**C**AAG**A TTATGTA**A**TC**A** | **-** | **-** |
| 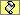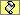 |  |  |  |  |  |  |
| **β-proteobacteria** |  |  |  |  |  |  |
|  |  |  |  |  |  |  |
| *Burkholderia cenocepacia* AU 1054 | 1 | 3294563 | 1347909 | AATGTCGATAA T**GTTGA** TTATGT**C**AATT | **+** | **+** |
|  | 2 | 2788459 | 1122591 | AA**G**GTCGATAA T**TCACG** TTATGT**A**AAAT | **-** | **-** |
|  | 3 | 1196094 | 1000473 | AA**T**GTCGATAA T**CAGCA** TTATGT**C**AAAT | **-** | **-** |
| *Burkholderia ambifaria* AMMD | 1 | 3556545 | 1814911 | **AACT**T**C**GATAA TT**ATGA** TTATGT**C**AAAT | **+** | **+** |
|  | 2 | 2646969 | 703451 | **AATG**T**T**GATAA TT**CACG** TTATGT**A**AAAT | **-** | **-** |
|  | 3 | 1281472 | 1203284 | **TCGG**T**T**GATAA TT**TACC** TTATGT**A**AAAT | **-** | **-** |
| *Burkholderia mallei* ATCC 23344 | 1 | 3510148 | 1081308 | AATGTCGATAA TT**GATA** TTATGTCAAAT | **+** | **+** |
|  | 2 | 2325379 | 1075134 | AATGTCGATAA TT**TGCG** TTATGTCAAAT | **-** | **-** |
| *Burkholderia pseudomallei* K96243 ^(3)^ | 1 | 4074542 | 1993992 | AATGTCGATAA TT**GATA** TTATGTCAAAT | **+** | **+** |
|  | 2 | 3173005 | 1462229 | AATGTCGATAA TT**TACC** TTATGTCAAAT | **-** | **-** |
| *Burkholderia thailandensis* E264 | 1 | 3809201 | 2649012 | AA**TA**T**T**GATAA TT**G**A**TA** TTATGTCAAAT | **+** | **+** |
|  | 2 | 2914771 | 1578744 | AA**GG**T**C**GATAA TT**T**A**CG** TTATGTCAAAT | **-** | **-** |
| *Burkholderia xenovorans* LB400 | 1 | 4895836 | 2500005 | **T**A**G**GTTGATAA TTTAT**A** TTATGTCAA**A**T | **+** | **+** |
|  | 2 | 3363523 | 1673941 | **T**A**T**GTTGATAA TTTAT**C** TTATGTCAA**G**T | **-** | **-** |
|  | 3 | 1471779 | 702059 | **C**A**T**GTTGATAA TTTAT**A** TTATGTCAA**A**T | **-** | **-** |
| *Ralstonia eutropha* JMP134 ^(4)^ | 1 | 3806533 | 1847779 | T**G**TCCGCATAA T**GC**GT**A** TTATGTTAAAT | **+** | **+** |
|  | 2 | 2726152 | 765636 | T**T**TCCGCATAA T**CG**GT**C** TTATGTTAAAT | **-** | **-** |
| *Ralstonia metallidurans* CH34 | 1 | 3928089 | 1892806 | AATAC**CA**ATAA T**C**TAT**C** TTATGTTAAAT | **+** | **+** |
|  | 2 | 2580084 | 947638 | AATAC**AT**ATAA T**G**TAT**A** TTATGTTAAAT | **-** | **-** |
| **γ-proteobacteria** |  |  |  |  |  |  |
|  |  |  |  |  |  |  |
| *Pseudoalteromonas haloplanktis* TAC125 | 1 | 3214944 | 1580520 | ACA**A**CC**T**ATAA **T**TTA**TA** TTATGTTAAAT | **+** | **+** |
|  | 2 | 635328 | 619766 | ACA**G**CC**C**ATAA **A**TTA**CG** TTATGTTAAAT | **-** | **-** |
| *Photobacterium profundum* SS9 | 1 | 4085294 | 2065371 | AGTGCGCATTA TGTA**TA** TTATGTTAAAT | **+** | **+** |
|  | 2 | 2237927 | 1074457 | AGTGCGCATTA TGTA**CC** TTATGTTAAAT | **-** | **-** |
| *Vibrio cholerae* O1 biovar eltor str. N16961 | 1 | 2961116 | 1564117 | A**G**TGCG**T**ATTA **T**GT**AT**G TTATGTTAAAT | **+** | **+** |
|  | 2 | 1072311 | 507997 | A**A**TGCG**C**ATTA **C**GT**GC**G TTATGTTAAAT | **-** | **-** |
| *Vibrio fischeri* ES114 | 1 | 2906169 | 1449378 | AGTGCGCATTA T**G**TAT**A** TTATGTTAAAT | **+** | **+** |
|  | 2 | 1332021 | 658896 | AGTGCGCATTA T**A**TAT**C** TTATGTTAAAT | **-** | **-** |
| *Vibrio parahaemolyticus* RIMD 2210633 | 1 | 3288558 | 1658763 | AGTGCG**C**ATTA T**G**T**A**T**A** TTATGTTAAAT | **+** | **+** |
|  | 2 | 1877212 | 933586 | AGTGCG**T**ATTA T**A**T**G**T**G** TTATGTTAAAT | **-** | **-** |
| *Vibrio vulnificus* CMCP6 ^(5)^ | 1 | 3281945 | 2661304 | **A**GTGCGCATTA TGTA**TA** TTATGTTAAAT | **+** | **+** |
|  | 2 | 1844853 | 313973 | **G**GTGCGCATTA TGTA**CG** TTATGTTAAAT | **-** | **-** |

^(1)^ Nucleotides in bold represent the differences between the *dif* sequences within the same strain. Underlined nucleotides correspond to the difference between strains within the same species

^(2)^ *dif*-related sequence identical to *Brucella melitensis* biovar Abortus 2308

^(3)^ *dif*-related sequence identical to *Burkholderia pseudomallei* 1710b

^(4)^ *dif*-related sequence different from *Ralstonia eutropha* H16

^(5)^ *dif*-related sequence different from *Vibrio vulnificus* YJ016
